# Microbiome sequencing revealed the abundance of uncultured bacteria in the Phatthalung sago palm-growing soil

**DOI:** 10.1371/journal.pone.0299251

**Published:** 2024-03-05

**Authors:** Pumin Nutaratat, Tantip Arigul, Nantana Srisuk, Worarat Kruasuwan

**Affiliations:** 1 Department of Biology, Faculty of Science and Digital Innovation, Thaksin University, Pa Phayom, Phatthalung, Thailand; 2 Microbial Technology for Agriculture, Food and Environment Research Center, Faculty of Science and Digital Innovation, Thaksin University, Pa Phayom, Phatthalung, Thailand; 3 Division of Medical Bioinformatics, Research Department, Faculty of Medicine Siriraj Hospital, Mahidol University, Bangkok, Thailand; 4 Siriraj Long-Read Lab (Si-LoL), Faculty of Medicine Siriraj Hospital, Mahidol University, Bangkok, Thailand; 5 Department of Microbiology, Faculty of Science, Kasetsart University, Bangkok, Thailand; 6 Biodiversity Center Kasetsart University (BDCKU), Bangkok, Thailand; Babasaheb Bhimrao Ambedkar University, INDIA

## Abstract

Environmental variations have been observed to influence bacterial community composition, thereby impacting biological activities in the soil. Together, the information on bacterial functional groups in Phatthalung sago palm-growing soils remains limited. In this work, the core soil bacterial community in the Phatthalung sago palm-growing areas during both the summer and rainy seasons was examined using V3-V4 amplicon sequencing. Our findings demonstrated that the seasons had no significant effects on the alpha diversity, but the beta diversity of the community was influenced by seasonal variations. The bacteria in the phyla Acidobacteriota, Actinobacteriota, Chloroflexi, Methylomirabilota, Planctomycetota, and Proteobacteria were predominantly identified across the soil samples. Among these, 26 genera were classified as a core microbiome, mostly belonging to uncultured bacteria. Gene functions related to photorespiration and methanogenesis were enriched in both seasons. Genes related to aerobic chemoheterotrophy metabolisms and nitrogen fixation were more abundant in the rainy season soils, while, human pathogen pneumonia-related genes were overrepresented in the summer season. The investigation not only provides into the bacterial composition inherent to the sago palm-cultivated soil but also the gene functions during the shift in seasons.

## Introduction

Peatlands are characterized as unique ecological environments resulting from the extended accumulation of organic matter derived from plants. In peatlands, year-round waterlogged conditions lead to a substantial reduction in the microbial decomposition rate of plant litter and organic matter, resulting in high carbon absorption and helping to mitigate the climate crisis [1]. Nevertheless, the processes of deforestation, drainage, and conversion of peatland to agricultural land are adversely affecting global carbon dioxide emissions by 5–10% [1–3]. Hence, the restoration of disturbed and degraded peatlands has emerged as a critical priority in efforts to address climate change.

In Southeast Asia, peatland ecosystems account for approximately 26 million hectares, mostly in Indonesia, Malaysia, Singapore, and Southern Thailand. There are approximately 63,800 hectares of peatland in Thailand that have been significantly used for palm oil plantations since the year 2000 [4]. Besides, sago palm (*Metroxylon sagu* Rottb.) is a carbohydrate-producing plant and one of the typical indigenous food crops in Southeast Asia, particularly Indonesia, which represents the largest sago palm-growing region [5, 6]. Moreover, the sago palm is also distributed across Malaysia, the Philippines, and the southern part of Thailand [7]. Sago palms are mostly grown well in peat swamp forest areas and also in very acidic to neutral soils, which is a potential resource for carbon sequestration in peat swamps, resulting in a lower rate of carbon dioxide emission and mitigating the impact of climate change on temperature fluctuations in the Earth’s climate system [8]. The sago palm is one of the plants that can grow optimally in waterlogged conditions or peatlands and is mainly found in the southern part of Thailand. Nowadays, the sago palm forest in Ban Hua Phru, Khuan Khanun, Phatthalung remained an area of approximately 11 out of a total of 32 hectares of peat soil. This forest plays a crucial role as a symbol for preserving the traditional landscape, ecosystems, sociocultural heritage, and biodiversity [9]. Moreover, the cultivation of sago palms on peat soil has been reported to offer benefits not only in water and soil conservation but also in maintaining the overall environmental integrity, leading to the mitigation of greenhouse effects [6]. Currently, the forests have been rapidly destroyed, and the planting areas are likely to decrease due to watercourse dredging by local and government agencies as well as severe climate changes.

Microorganisms, including archaea, bacteria, and fungi, predominantly participate in the degradation of soil organic carbon in peatlands. Their activities influence carbon sequestration by enhancing the rate of organic carbon degradation, thereby converting these peat soils into sources of carbon dioxide [10]. The examination of microbial abundance in peatland in Changbai Mountain, China, specifically considering soil depths and physicochemical properties, revealed a notable impact of soil depth on total microbial abundance rather than altitude. These findings suggest that variations in the environment directly influence microbial abundance in peat soils at depths of 5–10 cm [11]. On the one hand, an increase in temperature has been observed to rise the emission of carbon dioxide, driven by respiratory activity and microbial abundance in peat samples collected in La Guette peatland, France [12]. On the other hand, high-throughput sequencing technology demonstrated no substantial impact on the alpha diversity of the bacterial community during short-term warming, whereas the fungal community in the alpine peatland was influenced by soil moisture based on short-read amplicon sequencing of either 16S rRNA or ITS genes [13]. Thus, a complete understanding of the microbial communities in peatlands is crucial for unraveling the microbial dynamics and ecological processes in these ecosystems.

The emergence and application of high-throughput sequencing technologies have revolutionized the ability to explore microbial diversity, allowing for the direct investigation of bacteria in environmental samples and enabling researchers to explore the hidden microbial world beneath the soil surface. Together, the abundance and community structure of bacteria including the functional group in naturally grown sago palms remain poorly understood. Hence, in this study, peat soil samples from the sago palm forest in Phatthalung, Thailand collected during the summer and rainy seasons were used to determine the bacterial abundance using paired-end sequencing of the V3-V4 region of the 16S rRNA gene. This study sheds light on the richness, evenness of bacterial community and functioned genes analysis in this unique ecosystem.

## Materials and methods

### Study site and sampled soils

**Fig 1:**
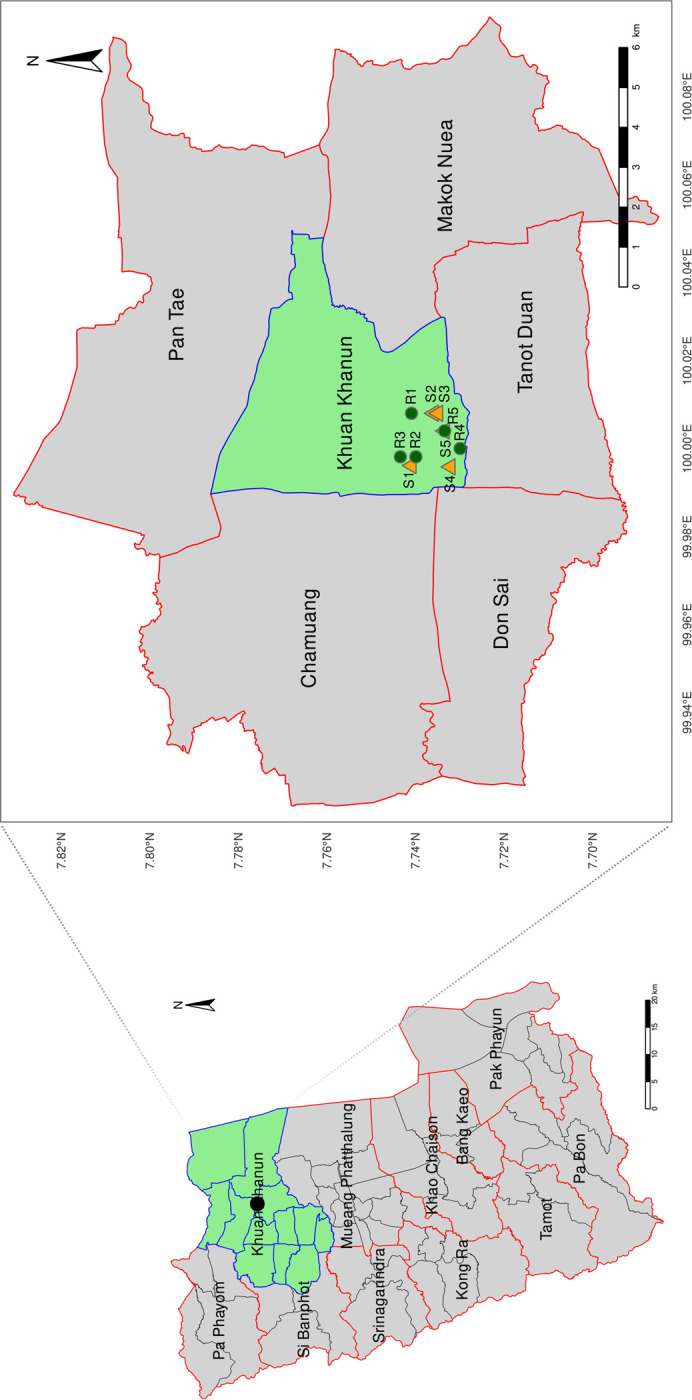
Five different sites of soil samples collected in the sago palm forest. Soil samples were collected at Ban Hua Phru, Khuan Khanun, Phatthalung, Thailand during the summer (orange triangle, S1 to S5) and the rainy seasons (green circle, R1 to R5).

Soil samples used in this work were collected from five different sites in the sago palm forest at Ban Hua Phru, Khuan Khanun, Phatthalung, Thailand (Fig 1 and S1 Table in S2 File). All five collected sites displayed the same soil type, which was peat soil containing alluvial sand and clay. Each location was sampled with a minimum distance of 300–500 meters from adjacent sites. Briefly, surface soils to 5–10 cm in depth (30 g per sample) were collected during the summer season (S1-S5) on March 2021 (*n* = 5) and the rainy season (R1-R5) on August 2021 (*n* = 5) with a sterilized spatula. The total of 10 soil cores (5 sampling sites × 2 seasons) were stored at –20°C until further analysis. Soil temperature and pH were measured from five independent replicates (*n* = 5). Soil moisture content was measured by drying each sample in a thermostatically controlled drying oven maintained at a temperature of 105 ± 5°C for 48 hours. The difference between the weight of the fresh sample and the weight of the air-dried soil sample was used as a proxy for soil moisture. Daily meteorological data (rainfall, rain precipitation, and humidity) was obtained from the Thai Meteorological Department of the Ministry of Digital Economy and Society of Thailand. No permits were required for the described study, which complied with all relevant regulations.

### DNA extraction and V3-V4 amplicon sequencing

Total genomic DNA was extracted from 250 mg soil using the DNeasy PowerSoil Pro DNA Kit (Qiagen, USA) following the manufacturer’s protocol. The DNA quantity was measured by a Nanodrop spectrophotometer (Thermo Fisher Scientific, USA). The 341F and 805R primers were used to amplify the V3-V4 region of the 16S rRNA gene for bacterial communities. PCR was processed in a 50 μl total volume by using 2× sparQ HiFi PCR master Mix (QuantaBio, USA) under the following conditions: initial denaturation for 2 min at 98°C; 28 cycles of denaturation for 20 sec at 98°C; annealing for 30 sec at 60°C; extension for 1 min at 72°C; and a final extension step for 1 min at 72°C. Subsequently, the amplicon was purified using sparQ Puremag Beads (QuantaBio, USA) and indexed using 5 μl of each Nextera XT index primer in a 50 μl PCR reaction using 10 cycles of the above described PCR conditions. PCR products were combined, purified using sparQ Puremag Beads (QuantaBio, USA), and pooled to produce a final loading concentration of 4 pM. Then, pooled libraries were sequenced using Illumina MiSeq (Illumina, USA) to generate paired-end reads in 2×250 bp using the 341F and 805R primers [14].

### Raw sequences pre-processing and bacterial taxonomically assignment

The V3-V4 amplicon sequences were analyzed using Quantitative Insights into Bacterial Ecology version 2 (QIIME2 v2020.8) using bacterial 16S processing workflows [15]. The raw paired-end reads were demultiplexed, adapter trimmed, and then quality filtered and denoised using the DADA2 (q2-dada2 plugin) [16]. The high-quality reads were further used to produce amplicon sequence variant (ASV) tables. Then, taxonomy classification was performed using q2-feature-classifier [17] by classifying the extracted reads against the SILVA v138 [99% OUT (operational taxonomic unit) reference sequences] database at a 97% similarity cut-off [18]. We calculated metrics of alpha and beta diversity using QIIME2, rarefying to 33,604 reads per sample at the time of metric computation. For alpha diversity, observed ASVs, the Shannon index, and Faith’s phylogenetic diversity were measured. Non-parametric Kruskal-Wallis tests were performed to compare the alpha diversity index [19]. For beta diversity, permutational multivariate analysis of variance (PERMANOVA) with two beta diversity metrics, Bray-Curtis dissimilarity matrix and Jaccard index, were analyzed in order to capture bacterial community distances between pairs of samples respectively based on the presence and relative abundance of unique taxa [20]. Principal coordinates analysis (PCA) was estimated using q2‐diversity after samples were rarefied (subsampled without replacement) to 33,604 sequences per sample. Functional Annotation of Prokaryotic Taxa (FAPROTAX v1.2.2) was used to assign ecologically relevant functions to species [21].

### Data availability

The raw sequencing data are available at the NCBI Sequence Read Archive (SRA) under BioProject PRJNA863387 with accession numbers SRR20709323 to SRR20709332.

## Results

### Meteorological data of soil sample sites

The meteorological data of the soil sample sites analyzed in this study were the temperature, rainfall, rain precipitation, and humidity. Overall, the temperature and humidity did not differ significantly across the soils collected in March (summer season) and August (rainy season). However, the amount of rainfall, rain precipitation, and soil moisture content during the rainy season was obviously higher than in the summer season. Additionally, there was no significant difference in soil pH between the soils collected during the summer and rainy seasons, with values ranging between 5.7 ± 0.35 and 5.9 ± 0.37, respectively (Table 1).

**Table 1:** Meteorological data and sago palm-growing soil properties collected in the summer and rainy seasons. Data are mean ± S.D. of five independent replicates (*n* = 5). Asterisk symbol represents the significant *P-*value (*P*<0.05).

Features	Summer season	Rainy season
	March 2021	August 2021
Air temperature (°C)	33.3 ± 0.45	32.6 ± 0.28
Rainfall (mm)	55.8 ± 0.70	137.2 ± 0.57*
Rain precipitation (%)	22.3 ± 7.63	50.8 ± 4.86*
Humidity (%)	99.0 ± 0.82	100.0 ± 0.00
Soil temperature (°C)	29.7 ± 1.35	29.0 ± 1.74
Soil moisture (g/g)	0.2 ± 0.12	0.6 ± 0.10*
Soil pH	5.7 ± 0.35	5.9 ± 0.37

### Soil microbiome data processing

To investigate the impact of seasonal variation on the bacterial community in the sago palm soil microbiomes, we collected five soil samples in March (summer season, referred to as S1 to S5) and August (rainy season, referred to as R1 to R5) from the Phatthalung sago palm forest (Fig 1 and S1 Table in S2 File). The microbial community analysis was conducted using paired-end sequencing of the V3-V4 region of the ribosomal 16S rRNA on the Illumina MiSeq platform (S2 Table in S2 File), resulting in a total of 1,109,839 reads (S3 Table in S2 File). The number of processed reads per sample ranged from 33,604 to 61,994. To minimize the bias introduced by differences in sample depth, all samples were rarefied to an even number of 33,604 reads prior to conducting diversity analysis (S1 Fig in S1 File).

### Soil microbial community composition

**Fig 2:**
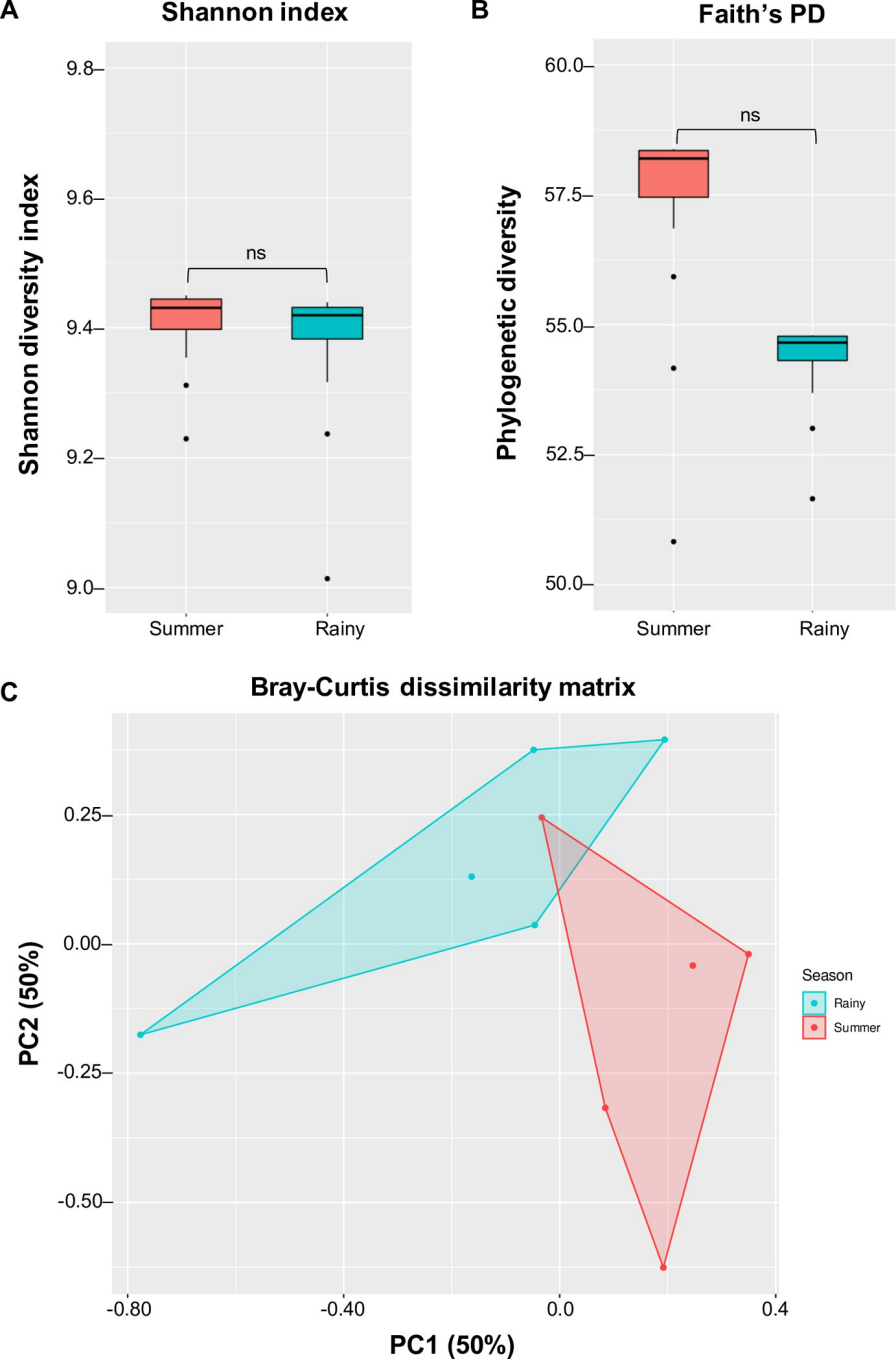
Microbial alpha and beta diversity in the soil samples collected in the sago palm forest. Boxplot displays (A) the Shannon index, (B) Faith’s phylogenetic diversity, and (C) principal coordinates analysis (PCA) of a Bray-Curtis dissimilarity matrix.

To examine the impact of seasonal variation on bacterial diversity, alpha diversity was calculated from soil samples collected during the summer and rainy seasons. The analysis of variance for bacterial richness revealed that soil samples collected during the summer season (1,040 ASVs) exhibited a slightly higher number of observed ASVs compared to the rainy season (979 ASVs). Among the samples, S1 displayed the highest number of ASVs (1,078), while R5 had the lowest (652) (S4 Table in S2 File). However, based on the Shannon index, which measures the richness of expected taxa within a sample, there were no significant differences among the soil samples (Fig 2A, Kruskal-Wallis H-test: *H* = 0.53, *P* = 0.46). Additionally, Faith’s phylogenetic diversity metric provided further evidence by indicating no significant degree of phylogenetic divergence in the summer samples compared to the rainy season (Fig 2B, Kruskal-Wallis H-test: *H* = 0.27, *P* = 0.60). To assess the influence of seasonal variation on community structure, a PCA plot based on the Bray-Curtis dissimilarity matrix was generated. The plot revealed that the soil microbiome data from the summer season formed a distinct cluster, suggesting a divergence in bacterial community composition between the summer and rainy seasons (Fig 2C, PERMANOVA: pseudo-*F* = 1.779, *P* = 0.01). Similarly, Jaccard index analysis (PERMANOVA: pseudo-*F* = 1.769, *P* = 0.03) further confirmed the significant influence of seasons on the beta diversity of the bacterial community.

### Common taxa in the growing sago palm soil

**Fig 3:**
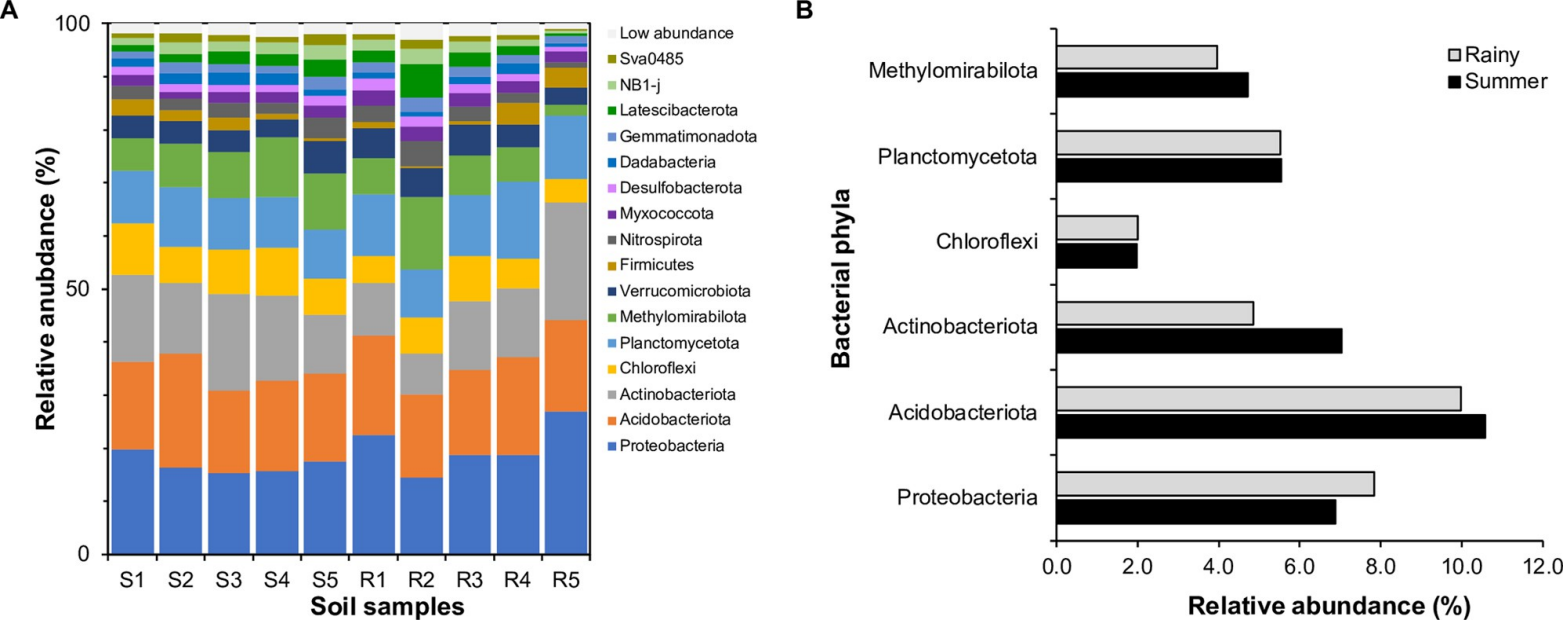
Taxonomic distribution and average relative abundance of the uncultured bacterial communities. (A) Distribution of bacterial taxonomic community and (B) and average relative abundance of uncultured bacteria (relative abundance ≥1%, *n* = 5) found in Phatthalung sago palm-growing soils collected during the summer (S1-S5) and rainy (R1-R5) seasons. Colour families display unique microbial phyla and distinct shades reflect microbial family. Low relative abundance refers to the summation of all taxa that failed to reach the 1% cut-off.

We evaluated the impact of seasonal variation on the bacterial community composition in the sago palm forest during the summer and rainy seasons. Our analysis revealed significant variations in the relative abundance of bacterial phyla within the soil communities, with a total of 16 phyla accounting for ≥1% of the relative abundance. The dominant phyla in the tested soil were Proteobacteria, Acidobacteriota, Actinobacteriota, Chloroflexi, Methylomirabilota, and Planctomycetota which collectively constituted 9–17.4% of the summer and 7.3–20.3% of the relative abundance of rainy collected soils (Fig 3A and S5 Table in S2 File). Among these phyla, relative abundance of Acidobacteriota was comparable in both the summer (17.4%) and rainy (17.2%) seasons. In contrast, Proteobacteria showed a 19% increase in relative abundance, while Methylomirabilota exhibited an 18% decrease in the rainy season (S5 Table in S2 File). Within the 16 phyla, we identified 31 genera in the soil samples collected during the summer season and 29 genera in the rainy season, with a mean relative abundance ≥1% (S6 Table in S2 File). A total of 26 putative core microbiomes were identified, representing bacterial genera present in all collected soil samples. The dominant bacteria within these core microbiomes belonged to the uncultured bacteria in the phyla Acidobacteriota (~10%) and Proteobacteria (~7%), followed by Actinobacteriota, Methylomirabilota, and Chloroflexi (Fig 3B), with some classified as *Bacillus* and *Gaiella* (S6 Table in S2 File). Furthermore, we found five uncultured bacteria in the phylum Acidobacteriota, Chloroflexi, Sva0485, and an uncultured Geminicoccaceae bacterium that were exclusively present in the soil collected during the summer season. While three genera (*Candidatus* Udaeobacter, *Pirellula*, and uncultured bacteria in the phylum Planctomycetota) were unique to the rainy season (S7 Table in S2 File).

### Functional annotation of soil microbiome

**Fig 4:**
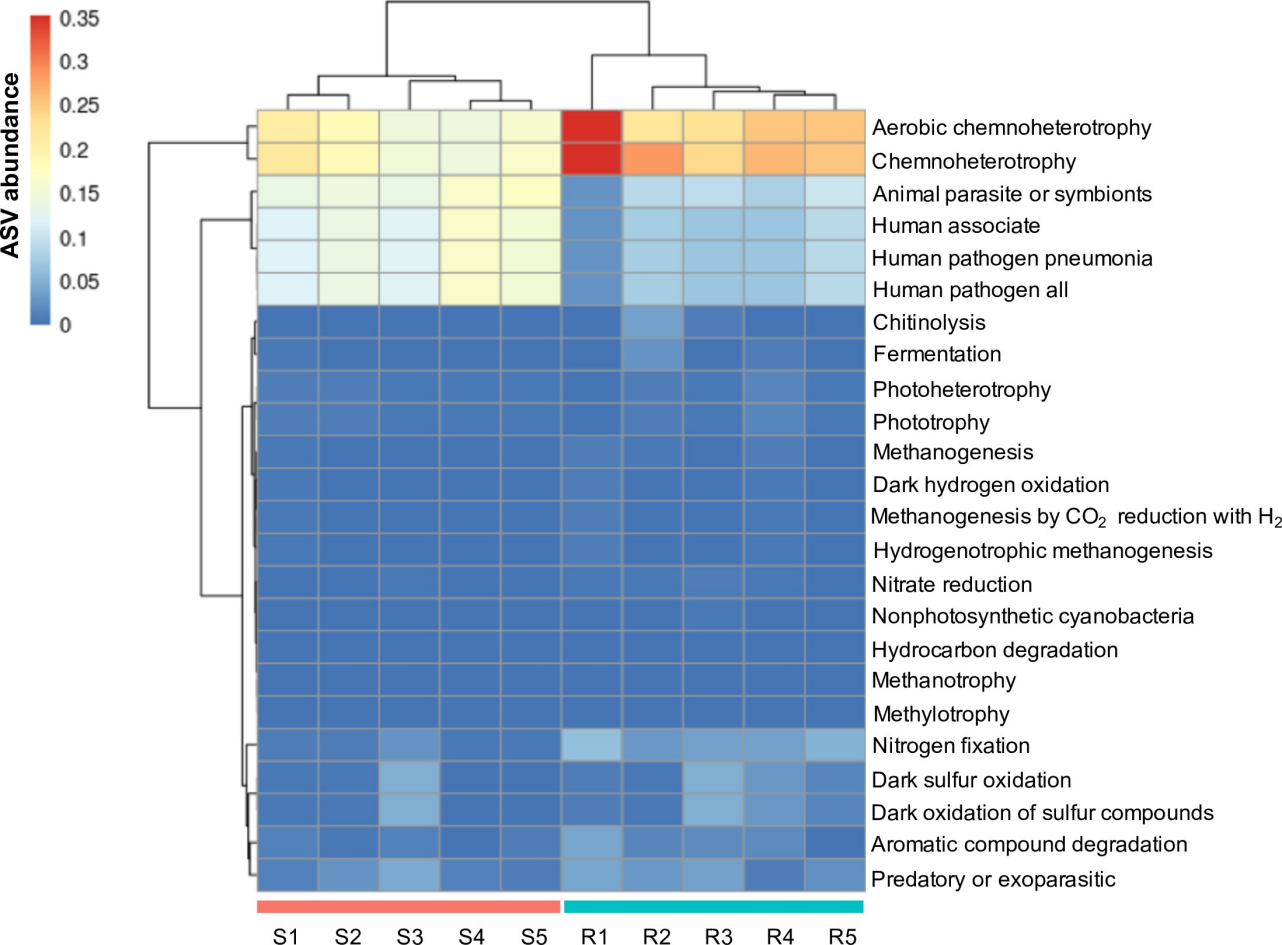
Functional characteristics of soil microbial communities collected in the sago palm forest. Heatmap of metabolic and ecological functions of bacteria based on FAPROTAX prediction. Data are based on ASVs abundance (number of ASVs capable of each function) in during the summer (S1-S5) and the rainy (R1-R5) seasons.

To assign functional properties to the identified core microbiomes in the sago palm-growing soils, we conducted a functional annotation using the FAPROTAX (Functional Annotation of Prokaryotic Taxa) analysis. This analysis allowed us to generate putative functional profiles of the microbial communities inhabiting the sago palm soil based on their community composition (Fig 4 and S8 Table in S2 File). Our annotation revealed the presence of 24 functional categories in the sago palm-growing soil microbial community. Gene involved in chitinolysis, fermentation, methogenesis, and photorespiration were commonly found in both the summer and rainy seasons. Several functions showed higher abundance in soils collected during the rainy season, including aerobic chemoheterotrophy, other chemoheterotrophy, nitrogen fixation, sulfur metabolism, and aromatic compound degradation. On the other hand, putative functions associated with animal parasites or symbionts, as well as the human pathogen pneumonia, were found to be overrepresented in soils collected during the summer season.

## Discussion

In this work, we investigated the profiling of soils utilized for sago palm cultivation in Phatthalung, Thailand, during the summer and rainy seasons using the 16S-amplicon microbiome technique. The core microbial profiling and functional gene analysis of these soil microbial communities across the seasons were characterized. In general, the soil microbiota exhibited comparable levels of diversity during both the summer and rainy seasons, indicating that seasonal variations did not significantly influence microbial profiling in the collected soils from sago palm-growing areas (Fig 2). The smaller observed ASVs in the rainy season may be due to the considerably higher rainfall and percentage of rain precipitation (S4 Table in S2 File). We found that the Acidobacteriota phylum was common abundant bacterial group found in both the summer and rainy seasons at the same proportion of approximately 17%, while Actinobacteriota constitute the key minor bacterial taxa (S5 Table in S2 File). However, during the rainy season, the Proteobacteria phylum remained the most prevalent group compared to the summer season, similar to findings that reported an increase in Proteobacteria and Firmicutes during the rainy season [22]. A previous report demonstrated that microbial communities in tropical peat swamp forests exhibit high levels of diversity and are primarily dominated by members of the Acidobacteriota phylum [23]. Similarly, our investigation revealed a predominant presence of this phylum in the sago palm soils (~17%), which corresponds with its relative abundance in Indonesian peat soils (32.6%), Brazilian peatlands (22–56%), and Narathiwat peat swamp forest [24–26]. Acidobacteriota has been identified as an essential bacterial taxon in soil ecosystems, playing a crucial role in the decomposition of soil organic matter and thus influencing carbon turnover dynamics [27, 28]. Consequently, the high relative abundance of Acidobacteriota in sago palm-growing soils can be attributed to the presence of substantial organic matter accumulation resulting from waterlogging and acidity, which contribute to the formation of peat.

Subsequently, the core microbiome in the tested soil was classified at the genus level to explore the microorganisms that inhabit the sago palm-growing soils of Phatthalung. Our results found that the core microbiota was mostly attributed to uncultured bacterial taxa of the Acidobacteriota, Proteobacteria, Actinobacteriota, Planctomycetota, Methylomirabilota, and Chloroflexi members, respectively (Fig 3B). The high prevalence of those uncultured bacteria was found to be crucial in its ecology as they have been classified as either producers of polysaccharide-degrading enzymes or as microbes involved in the degradation of plant biomass in the peat swamp forest [29]. Functional prediction of the microbial community confirmed the presence of those bacteria as a core microbiome by displaying gene functions related to chitonolysis and hydrocarbon degradation, for instance, in both seasons of the sago palm-growing soils of Phatthalung. Furthermore, the core microbiota exhibited a notable abundance of genes associated with methanogenic bioprocesses (methanogenesis, methanotrophy, methylotrophy, hydrogenotrophic methanogenesis, and methanogenesis by CO_2_ reduction to H_2_) (Fig 4). *Candidatus* Methanoperedens nitroreducens and *Candidatus* Methylomirabilis oxyfera, both classified under group A of the Methylomirabilota phylum, have been identified as key players in the nitrogen and methane cycles [30]. Moreover, an increased temperature found to enhance the CH_4_ oxidation potential in sediments of Fildes Peninsula freshwater lakes, King George Island, Antarctica [31]. Therefore, the existence of these methane-associated genes and bacteria within the Methylomirabilota phylum demonstrated the prevalence of methanotrophic bacteria within the sago palm forest and the findings are comparable to functional predictions of uncultured bacteria in peat soil [31, 32]. However, further investigation is necessary to elucidate the interactions of these uncultured bacteria within methanogenic environments. As has been shown in this study, the microbial communities in the sago palm-growing soils of Phatthalung were diverse and abundant with uncultured bacteria. However, some of them were characterized as *Bacillus* and *Gaiella* genera (S7 Table in S2 File) which agrees with a previous report indicating the presence of *Bacillus* and *Lysinibacillus* in the rhizosphere soil of Malaysian sago palm, potentially enhancing plant growth through the production of plant growth-promoting agents [33].

Rainfall can influence bacterial diversity by increasing water levels, thereby enriching bacteria belonging to the *Candidatus* Udaeobacter and *Pirellula* genera (S7 Table in S2 File). In German forest soil ecosystems, *Ca*. Udaeobacter was found to be predominantly abundant. Genome analysis of *Ca*. Udaeobacter has revealed a diverse collection of antibiotic resistance genes and hydrogenase genes, indicating its potential involvement in H_2_-based energy generation [34]. Additionally, in the anoxic peat layers of acidic northern wetlands in Russia, a highly abundant strain of *Pirellula*-like planctomycetes has been identified [35]. Several uncultured bacteria, including the uncultured Geminicoccaceae bacterium, were exclusively detected during the summer season (S7 Table in S2 File). This bacterium, classified under the Proteobacteria phylum, has been found to inhabit desert niches in China and produce the plant growth hormone, indole-3-acetic acid. Genome analysis of this bacterium has revealed the presence of stress-tolerant associated genes and secondary metabolite gene clusters, which are believed to significantly contribute to their ability to survive in challenging desert environments [36]. Furthermore, a notable overrepresentation of gene functions associated with human pathogens linked to pneumonia and human associate was observed in the soil samples collected during the summer season. This observation can be attributed to the heightened human activity in the sago palm-growing areas, particularly during the months of January through March, when the harvest of the sago palm is commonly conducted (Fig 4 and S8 Table in S2 File).

## Conclusion

This study sheds light on the microbial profiling within the sago palm-growing areas of Phatthalung during both the summer and rainy seasons. Our analysis reveals no significant seasonal effects on alpha diversity, yet discernible shifts in beta diversity due to changing seasons. The core microbiome of the tested soils comprised predominantly uncultured bacteria, and functional gene predictions suggest their involvement in distinct functional roles within the Phatthalung sago palm-growing ecosystem. Owing to the challenges associated with culturing these bacteria, however, further investigations are necessary to elucidate their contributions to either carbon, methane transformations, or plant growth promotion in peat soil systems.

## Supporting information

S1 File Supporting Fig. (DOCX)

S2 File Supporting tables. (XLSX)
