# Supplementary material for: Microbiome sequencing revealed the abundance of uncultured bacteria in the Phatthalung sago palm-growing soil
Source: PLoS One. 2024 Mar 5;19(3):e0299251. doi: 10.1371/journal.pone.0299251 (PMC10914256; doi:10.1371/journal.pone.0299251)
Supplement: S1 File — (DOCX) [file pone.0299251.s001.docx]

**Supplemental File**

**Microbiome sequencing revealed the abundance of uncultured bacteria
in the Phatthalung sago palm-growing soil**

Pumin Nutaratat^1,2^, Tantip Arigul^3,4^, Nantana Srisuk^5,6^_,_ and Worarat Kruasuwan^3,4,*^

^1^Department of Biology, Faculty of Science and Digital Innovation, Thaksin University, Pa Phayom, Phatthalung, Thailand

^2^Microbial Technology for Agriculture, Food and Environment Research Center, Faculty of Science and Digital Innovation, Thaksin University, Pa Phayom, Phatthalung, Thailand

^3^Division of Medical Bioinformatics, Research Department, Faculty of Medicine Siriraj Hospital, Mahidol University, Bangkok, Thailand

^4^Siriraj Long-read Lab (Si-LoL), Faculty of Medicine Siriraj Hospital, Mahidol University, Bangkok, Thailand

^5^Department of Microbiology, Faculty of Science, Kasetsart University, Bangkok, Thailand

^6^ Biodiversity Center Kasetsart University (BDCKU), Bangkok, Thailand

*** Correspondence author:**

Worarat Kruasuwan, Ph.D.

Email: worarat.kru@mahidol.edu (WK)

**S1 Fig.** The rarefaction curve between observed ASVs and sample depth displayed nearly plateau at 33,604 reads.
